# The Present Situation of the D.D.S.

**Published:** 1904-11

**Authors:** 


### Domestic Correspondence.

### THE PRESENT SITUATION OF THE D.D.S.

THREE RIVERS, CANADA, October 13, 1904.

TO THE EDITOR:

SIR,—Enclosed please find statement requested of me by several college men. It will serve the best interests of all concerned to publish same at an early date. The exhibits referred to I shall gladly furnish also.

There should be prompt and pronounced action by the colleges and it might be wise to invite me to the meeting and allow me to enter into the council.

Very respectfully,

J. H. WORMAN.

The German government has been instructed by their consuls that any three persons may obtain a charter and found a college or professional school, that their founders may be wholly unqualified, morally or intellectually, to conduct such an institution, and that there is *no supervision on the part of the State* to insure the reputable conduct of manager, preceptor, or student, and that therefore the American Academic honors are questionable ones. I refer especially to the reports of Consuls Bopp and Wever. (See Exhibits A to E.)

They have also reported that political influences prevail in the schools and in the State boards, and that while the dental schools now belonging to the National Association of Dental Faculties may be regarded as "reputable," there is after all no guarantee that they will remain so, for "changes are in America always to be looked for." (See Exhibit .)

Erich Richter, who at one time carried great influence in the German courts as an Expert, although now retired, still uses every opportunity to call in question the character of our institutions, and for the purpose of saving the Huxmanites from their just deserts has even belittled my standing as a consul, leaving the impression on German judges that I am only a political creature and in nowise comparable with a general consular officer. Richter has testified under oath that Huxman's G. A. D. S. of Chicago was reputable in 1890–93, and recognized by the Illinois Board of Dental Examiners, although the minutes of the board do not permit any such statement. (See Exhibit .) Richter may have been given a hearing in the Imperial foreign office, and his statements should have been contradicted everywhere.

Professor Dr. Miller, of whom the Americans are justly proud, aimed some years ago to give the Germans a better comprehension of American dental surgery, but did, unwittingly no doubt, more harm than good. Although he recognized the worth of the National Association of Dental Faculties, he weakened their standing by the statement that the smaller schools do not observe their obligations like the stronger ones, and by the imputation that only in a few colleges were the conditions for admission comparable to the preparation in Germany. The German minds cannot grasp the situation so indefinitely stated, and false impressions now prevail. He also repeated the unfortunate statement printed in the report of the United States Commissioner of Education in 1899–1900 regarding the many aliases of our swindling colleges, so that a German unfamiliar with the facts must conclude that we have hundreds of swindling institutions, scholastic and professional, although the facts are that we never had a score of them, and that at the present hour none exist; at least, none are operating successfully.

Professor Miller bestowed much praise on me, and, therefore, this criticism on his course must seem ungrateful to those who do not realize that I seek first, last, and always, simply the suppression of an unlawful traffic in American academic honors and the just treatment of American interests; that I would go to any length to suppress the impostor and traducer, and would not hesitate to combat any attempt to belittle our good name, even though it might for the moment work greatly to our disadvantage. It is our duty to uproot the evil done in our name abroad and bring to justice all who have imposed on foreign governments; thus, and thus only, may we hope to prove ourselves worthy of confidence and our institutions of recognition.

There is a kindly feeling for America in the educated and beaurocratic ranks of Germany, and we could easily obtain an international standing for the dental profession if the States of the Union would legislate on the lines of New York, or the Philippines.

By the law of reciprocity, when once adopted everywhere at home, we would provide further means to effect through diplomatic channels the fullest acknowledgment for our schools, not as private foundations for personal gain, but as institutions conducted *pro bono publico,* bearing the stamp of State authority, also a *quasi* recognition from the national government.

The various states of Germany have issued edicts for regulating the use of titles in their states and territories as the central government of Germany, that is to say, the imperial German government cannot interfere in church and school matters, the autonomous rights prevailing in the individual states.

Much variability has existed, therefore, in Germany as to the use of foreign academic titles and the values of foreign academic degrees. In some states the edicts, while apparently establishing the manner in which holders of such foreign academic honors might obtain permission for their use of the respective authority of the state, that is to say, of the minister of the interior and cultus, seem to have been merely intended to do away with former usage without the direct declaration against any foreign titles, for in recent years all petitions outside of Bavaria by the holders of American academic degrees have been unfavorably replied to.

The unfavorable decisions are, however, largely due to the fact stated above,—viz., that the German governments have come to look upon our academic and professional schools as affording no guarantee as to the worth of the academic honors conferred by them.

The German courts have also differed widely in their decisions regarding the rights of the holders of American academic honors who are desirous of using their titles in public life, and, as the title plays a most important part in European professional life, any abrogation of the practitioner's rights in this connection means a considerable curtailment of his income, if not the ruin of the practitioner.

Wherever the courts have been duly informed by me of the *bona fide* of our reputable institutions, the courts have ruled favorably, despite the fact that the prosecution was instituted by the state's attorney upon the direct allegations, or, still worse, instigations, of German practitioners.

As late as last August an appeal taken in the kingdom of Bavaria by a graduate of the Maryland Dental College from the ruling of a district court, where local prejudices had resulted in an unfavorable verdict, the higher court ruled the practitioner holding an American reputable degree entitled to its use, and I believe that with proper counsel and truthful representations an appeal to the still higher courts would result no less favorably.

The statement originally given out by Richter, and widely circulated and published in the professional and general press, that the imperial supreme court of Germany had rendered a decision against the use of the American academic title, the "Doctor of Dental Surgery," is groundless, as the ruling was on the title "Doctor Chirurgiæ Dentariæ," the supreme court holding that the American institutions having *private* foundation could not confer titles in other than the language of the land, and that Latin titles should emanate only from institutions of the State with concurrences of the same.

My efforts aim to effect a twofold result,—viz.:

1. An harmonious legislation in the States of our Union making the licenses exchangeable and the diploma a conferment of the school and profession (*i.e.*, the Board of Examiners).

2. An international comity in the profession according to the American State licenses and academic honors of a recognized (reputable) college the same recognition abroad that we accord to their university honors and state approbations.

